# Impact of injectable HAE on-demand treatments on health-related quality of life: a patient and caregiver interview study

**DOI:** 10.1186/s13223-025-00997-w

**Published:** 2025-11-29

**Authors:** Patrick F. K. Yong, Timothy J. Craig, Paula J. Busse, Tomaz Garcez, Rebekah Hall, Siu Hing Lo, Caleb Dixon, Paul K. Audhya, Alice Wang, Aleena Banerji, Sorena Kiani-Alikhan

**Affiliations:** 1 Clinical Immunology and Allergy, Frimley Health NHS Foundation Trust, Frimley, UK; 2 Penn State University, Hershey, PA USA; 3 Vinmec International Hospital, Times City, Hanoi, Vietnam; 4 VinUniversity, Hanoi, Vietnam; 5 Mount Sinai School of Medicine, New York, NY USA; 6 Immunology Department, Manchester University NHS Foundation Trust, Manchester, UK; 7 Acaster Lloyd Consulting Ltd, London, UK; 8 KalVista Pharmaceuticals, Inc., Framingham, MA USA; 9 Department of Medicine, Division of Rheumatology, Allergy, and Immunology, Massachusetts General Hospital, Boston, MA USA; 10 Harvard Medical School, Boston, MA USA; 11 Department of Immunology, Royal Free London NHS Foundation Trust, Pond St, London, NW3 2QG UK

**Keywords:** Hereditary angioedema, Health-related quality of life, Caregivers, Patients, Adolescents, On-demand treatment

## Abstract

**Background:**

Hereditary angioedema (HAE) attacks can be unpredictable, painful, and debilitating. Although studies described the burden of prophylactic HAE treatments on patient and caregiver health-related quality of life (HRQoL), few have explored the effects of injectable on-demand treatments on HRQoL.

**Objective:**

To understand the impact of injectable on-demand HAE treatments on HRQoL.

**Methods:**

Patients (aged ≥ 12 years) with ≥ 1 HAE attack in the prior 6 months and adult caregivers (aged ≥ 18 years) of patients of any age with HAE were recruited between July and October 2024 to complete a qualitative interview. Questions concerned on-demand injectable treatment use, impacts, and burden. Thematic analysis was used to identify key themes across responses.

**Results:**

The 25 study participants from the US and UK (17 patients; 8 caregivers), who completed the interview, highlighted emotional and logistical reasons for delaying or forgoing injectable on-demand treatment, including fear of needles, portability, and complexity of administration. All participants described at least one negative impact on HRQoL, including anxiety and pain associated with treatment administration, disruption of daily activities or work/school days, and impacts on personal relationships. Adolescent patients reported greater impacts than adult patients. Although indicated for self-administration, some adult and all adolescent patients reported needing assistance with administration of their injectable on-demand treatment. All participants expressed interest in an oral on-demand treatment for reasons including portability, pain-free administration, and ability to treat attacks earlier.

**Conclusion:**

This study highlights the unmet need for an on-demand treatment that allows for earlier, pain-free administration, ultimately increasing patient independence and improving HRQoL for both patients and caregivers.

**Supplementary Information:**

The online version contains supplementary material available at 10.1186/s13223-025-00997-w.

## Introduction

Hereditary angioedema (HAE) is a rare genetic disorder with an estimated prevalence of 2.6 per 100,000 people in the United States (US) and a minimum prevalence of 1 per 59,000 people in the United Kingdom (UK) [1, 2]. Characterized by unpredictable, painful, and debilitating attacks of skin and/or submucosa, HAE causes swelling in the extremities, face, gastrointestinal tract, and/or larynx [3]. These attacks, which can range from mild to very severe and are potentially life threatening if affecting the larynx [3], have negative effects on health-related quality of life (HRQoL) and activities of daily living [4–11].

Currently, there are three treatment strategies for the management of HAE—on-demand treatment of attacks (all-patients), short-term prophylaxis, and long-term prophylaxis—the latter two in appropriate patients. On-demand treatments have been shown to be effective at reducing the severity and duration of an attack [12–19]. HAE guidelines recommend the use of on-demand treatment be considered for all attacks, regardless of location or severity; the treatment of attacks as soon as is feasible after attack onset; and the carrying of on-demand treatment for the management of two attacks [20–22]. Short-term prophylaxis is recommended before medical, surgical, or dental procedures and other attack-triggering events to reduce the risk of a subsequent attack [20–22]. Long-term prophylaxis is prescribed to reduce attack frequency, but its continuous effectiveness relies on routine monitoring of dosing. Even when long-term prophylaxis is used as prescribed, patients can still have attacks [23–27].

Until July 2025 [28, 29], all approved on-demand treatments were administered as either an intravenous (IV) infusion (plasma-derived C1 inhibitor [C1INH] [15, 16, 19] and recombinant C1 inhibitor C1INH [17, 18]), or subcutaneous (SC) injection (icatibant, ecallantide [US only]) [12–14]. With parenteral on-demand therapies, many patients delay or completely forego treating their attacks due to various emotional, logistic, and clinical challenges they face when using these self-injectable, on-demand treatments [23, 30, 31]. Emotional challenges expressed by patients include stress and depression due to the unpredictability of attacks, anxiety related to pain of injections, embarrassment of carrying treatment, and fear of needles. Other deterrents include issues with the transport/portability of the treatment, venous access, and dexterity to self-treat at the time of the attack, as well as injection site reactions [23, 30, 32–34].

The challenges associated with injectable on-demand treatments also impact caregivers. Studies of caregiver burden have largely focused on prophylactic treatments for HAE (i.e., evidence on caregiver burden associated with on-demand medication use and administration is limited) [35–37]. However, caring for patients with a rare disease is known to take an emotional toll and to cause stress [36, 38–40]. Because of the genetic nature of HAE, this burden is compounded when caregivers are themselves living with HAE [35, 38].

To gather insights from both patient and caregiver perspectives, this study employed qualitative interviews to describe: (1) the impact of different types of injectable on-demand treatments on HRQoL, (2) the experiences with injectable on-demand treatment, and (3) the perceptions of a potential oral on-demand therapy.

## Methods

### Study design and participant recruitment

Five subpopulations were recruited between July and October 2024 using a soft quota of five participants in each group: adult patients with HAE, adolescent patients with HAE, patient-caregivers (adults with HAE who care for one or more people with HAE), caregivers of adults with HAE, and caregivers of children with HAE. Eligible patients had a self-confirmed diagnosis of HAE Type 1 or Type 2, ≥ 1 HAE attack in the past 6 months, previous experience using injectable on-demand treatments for HAE attacks, and were adults (≥ 18 years of age) or adolescents (12–17 years of age). Eligible caregivers were aged ≥ 18 years and were a primary caregiver for a patient with HAE Type 1 or Type 2 aged ≥ 18 years (‘caregiver of adult’) or aged < 18 years (‘caregiver of child’). Patient-caregivers met the inclusion criteria of both the patient and caregiver (to an adult or child) subpopulations (patient-caregiver data are included only in the “Patient” subgroup).

Participants were recruited through the internal databases of the patient advocacy groups Hereditary Angioedema Association (HAEA) for the US participants and HAE International (HAEi) for the UK participants. Eligible participants were assigned a unique identification number and provided a survey link that shared further information about the study, and interested participants provided informed e-consent before taking part. Adolescent participants were provided with an age-appropriate version of the information sheet and completed an assent form, and a parent or guardian completed a corresponding consent form. Adolescents were given the option of having a chaperone such as a parent or guardian present during the interview in an observer-only capacity. All participants received a one-time remuneration for their time and participation in the study. This study was reviewed and approved by the WCG Institutional Review Board (Cary, NC, USA).

### Interview structure

Interviews were conducted by experienced qualitative researchers and followed a semi-structured interview guide. Sections of the interview completed by participants depended on the subpopulation to which they belonged.

All participants completed the concept elicitation (CE) section during which they were asked open-ended questions to understand on-demand treatment use, impacts, and burden. Different interview questions were prioritized throughout data collection to ensure that the full range of topics intended to be explored were captured; therefore, not all participants were asked all questions in the interview guide. After the completion of approximately half of the interviews, further interviews with adult patients prioritized survey cognitive debriefing.

Using a visual analogue scale (VAS; 0 [no symptoms] to 100 [worst symptoms ever]), most adult patients and patient-caregivers were asked to rate “How are you feeling today?” (baseline) and then to rate “mild,” “moderate,” “severe,” and “very severe” attacks on the scale, and to complete the EuroQol-5 domain (EQ-5D) quality of life measure according to the following three scenarios: “feelings at time of the interview (current HRQoL),” “expectations if there were access to an oral on-demand treatment,” and “feelings during their or their care-recipient’s last HAE attack.”

### Analysis

Based on professionally transcribed interviews, CE data were analyzed using thematic analysis to identify key themes and subthemes regarding on-demand treatment use, impacts, and burden following the Braun and Clarke approach [41]. “Patient” data refer to information shared by an adult/adolescent patient or patient-caregiver; and “care-recipient” data refer to information shared by a caregiver about their patient. Reported counts combined prompted and unprompted mentions of concepts throughout the interviews. VAS responses were summarized using descriptive statistics; statistical comparisons were not performed due to sample size. Index scores for EQ-5D valuation data were calculated using a mapping function between the EQ-5D-5 level (EQ-5D-5L) and EQ-5D-3 level (EQ-5D-3L) reflecting US preference weights and were summarized using descriptive statistics; statistical comparisons were not performed due to sample size.

## Results

### Study participants

Interview participants included 25 individuals residing in either the UK (*n* = 8) or US (*n* = 17) (Table 1). Seventeen participants were classified as patients (12 adults, including 5 patient-caregivers, and 5 adolescents) and eight as caregivers (4 to adults and 4 to children). Most participants were White (80%) and female (76%). The mean age of caregivers and adult patients were similar (43 and 42 years, respectively), and the mean age of adolescent patients was 14 years (range 12–16).

**Table 1:** Demographic and clinical characteristics

Characteristics	Patients (*n* = 17)	Caregivers (*n* = 8)
**Demographics of interview participants**		
*Country of residence, n (%)*		
US^a^	11 (65)	6 (75)
UK^b^	6 (35)	2 (25)
*Age, y*		
Mean (SD)	34 (16.1)	43 (7.2)
Range	12–60	33–52
*Sex, n (%)*		
Female	12 (71)	7 (88)
Male	5 (29)	1 (12)
*Race, n (%)*		
White	13 (77)	7 (88)
Black or African American	1 (6)	1 (12)
Asian	1 (6)	0 (0)
Prefer not to answer	2 (11)	0 (0)
**Clinical characteristics of individuals with HAE**^c^		
*Time since last attack, d*		
Mean (SD)	35 (54.6)	71 (86.0)
Range	1–180	0–270
*Location of last attack, n (%)*^*d*^		
Abdomen	9 (53)	3 (38)
Extremities/ limbs	5 (29)	4 (50)
Face	2 (12)	2 (25)
Other	1 (6)	0 (0)
*Severity of last attack, n (%)*		
Mild	4 (24)	2 (25)
Moderate	7 (41)	4 (50)
Severe	6 (35)	2 (25)
*Attacks in past year, n*		
Mean (SD)	8 (7.0)	7 (6.2)
Range	1–20	1–20
*Time since diagnosis, y*		
Mean (SD)	21 (15.2)	10 (9.4)
Range	3–47	5–31
*LTP use, n (%)*		
Lanadelumab-flyo^e^	5 (28)	3 (38)
C1INH subcutaneous [human]^f^	3 (18)	2 (25)
Berotralstat^g^	3 (18)	0 (0)
Other	3 (18)	1 (12)
None reported	3 (18)	2 (25)

C1INH, C1 inhibitor; HAE, hereditary angioedema; LTP, long-term prophylaxis; SD, standard deviation; UK, United Kingdom; US, United States

^a^From the US, there were 11 patients: 7 adults, including 4 patient-caregivers, and 4 adolescents; and 6 caregivers: 3 of a child and 3 of an adult

^b^From the UK, there were 6 patients: 5 adults, including 1 patient-caregiver, and 1 adolescent; and 2 caregivers: 1 of a child and 1 of an adult

^c^Clinical data were self reported for adult patient, patient-caregiver, and adolescent patient participants. Clinical data were proxy reported for their care-recipient participants by their caregivers

^d^Cumulative percentages do not equal 100, as categories were not mutually exclusive

^e^Takhzyro

^f^Haegarda

^g^Orladeyo

**Fig. 1:**
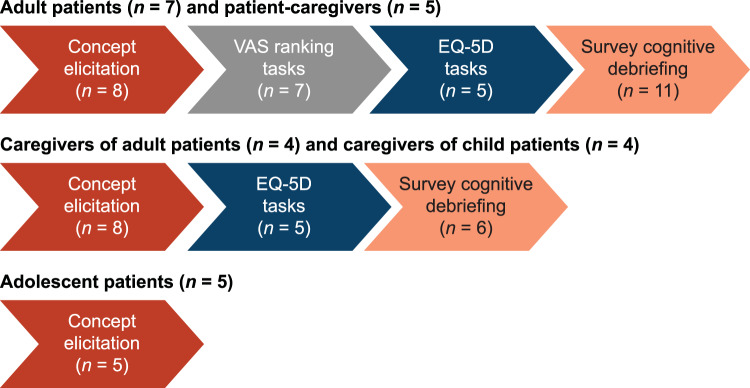
Summary of participants and sections of the interview guide covered during interviews. 5D, 5 domain; EQ, EuroQoL; VAS, visual analog scale

**Fig. 2:**
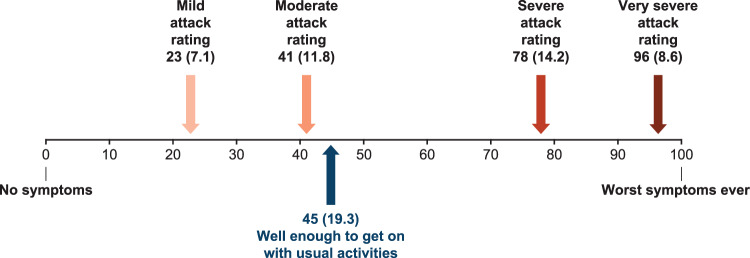
Qualitative VAS rating-task results among adult patients (*n* = 7), mean (SD). SD, standard deviation; VAS, visual analog scale

Figure 1 provides a summary of the participants completing each component of the interview. Most patients’ and care-recipients’ last attack was in the abdomen or limbs/extremities, and according to both patient and caregiver reports, most attacks (19/25, 76%) were categorized as moderate to severe (Table 1). VAS ratings for attack severity descriptions, provided in Fig. 2, illustrate that escalation in VAS pain ratings with increases in severity, with a mean rating of 23 for mild attacks and up to 96 for very severe attacks. Patients reported feeling well enough to perform activities of daily living with a mean VAS rating of 45, just above the mean rating of 41 for moderate attacks.

### On-demand treatment use and perceptions

On-demand treatment use and administration reported during interviews are shown in Table 2. As their primary on-demand treatment, 11 patients and 3 caregivers reported using icatibant (SC), while 6 patients and 5 caregivers reported using an IV C1INH. Eight patients (57%) self-administered icatibant, while six (43%) required a parent or caregiver to administer, including two of the 10 adult patients (20%) and all adolescents (*n* = 2). The three adolescents using C1INH had their injectable on-demand treatment administered in a health care setting.

**Table 2:** Typical on-demand treatment use

Characteristic of use, *n*	Icatibant (SC)	C1 inhibitor (IV)^a,b^
*Primary treatment*	14	11
Patients	11	6
Caregivers	3	5
*Administration*		
Self (adults only)^c^	8	4^d^
Caregiver/family member	6	1^d^
Health care professional	0	7
*Frequency of use*		
Treats every attack/suspected attack	4	1
Does not treat every attack	10	10
*Location of administration*		
Home only	9	1
Home or away from home	2	2
Hospital/medical facility	0	7
Not described	3	1

C1INH, C1 inhibitor; IV, intravenous; SC, subcutaneous

Counts include unprompted and prompted mentions. Counts include self-reported responses by patient participants and proxy-reported responses by caregiver participants and could, therefore, have resulted in double counting of reports from patients and caregivers related to each other

^a^On-demand plasma-derived C1INH for all participants except one adult and one adolescent respondent who had been prescribed recombinant C1INH

^b^All of the UK caregivers to children and the one adolescent patient from the UK reported using a C1INH

^c^No adolescents reported self-administration

^d^Combination of self-administration and administration by spouse

Most participants reported choosing not to treat every attack (10/14 of icatibant users and 10/11 of C1INH users). All participants reported delaying or forgoing on-demand treatment for attacks at least once in the past. Primary reasons for delaying or forgoing on-demand treatment centered around treatment issues, accessibility issues, attack features, and logistical factors (Supplemental Table 1).

Participants reported a dislike of needles, pain associated with injection of the medicine, and the need to go to a hospital or medical center as treatment-related reasons for delaying or opting not to treat an attack. Availability of treatment at medical facilities and challenges with insurance coverage created accessibility barriers for some participants.

Many participants opted not to treat attacks with milder symptoms or where swelling did not appear to be spreading to other anatomic locations. Some participants tended to wait until they were certain the symptoms were due to an attack rather than another cause, with delays due to symptom ambiguity mostly described in relation to abdominal attacks. However, most reported that they almost always treated facial, throat, and abdominal attacks, as these attack locations resulted in greater impacts on daily activities and the risk of life-threatening complications compared with other attack sites (e.g., hands, feet). Patients described feeling generally comfortable with self-administration but in some instances were unable to do so and needed assistance because of the location of swelling, e.g., of the hands, which prevented holding and using needles.

Participants (*n* = 19) also reported logistical reasons for delaying treatment including planned activities (e.g., would treat sooner if they had an important event or work), being away from home at the time of the attack and not carrying treatment, or being away from a caregiver who helps administer treatment.

These delays reportedly affected the efficacy of on-demand treatment, with some participants (*n* = 10) reporting increases in severity and duration of attacks (Supplemental Table 1).

### Perceptions of injectable on-demand treatments

**Fig. 3:**
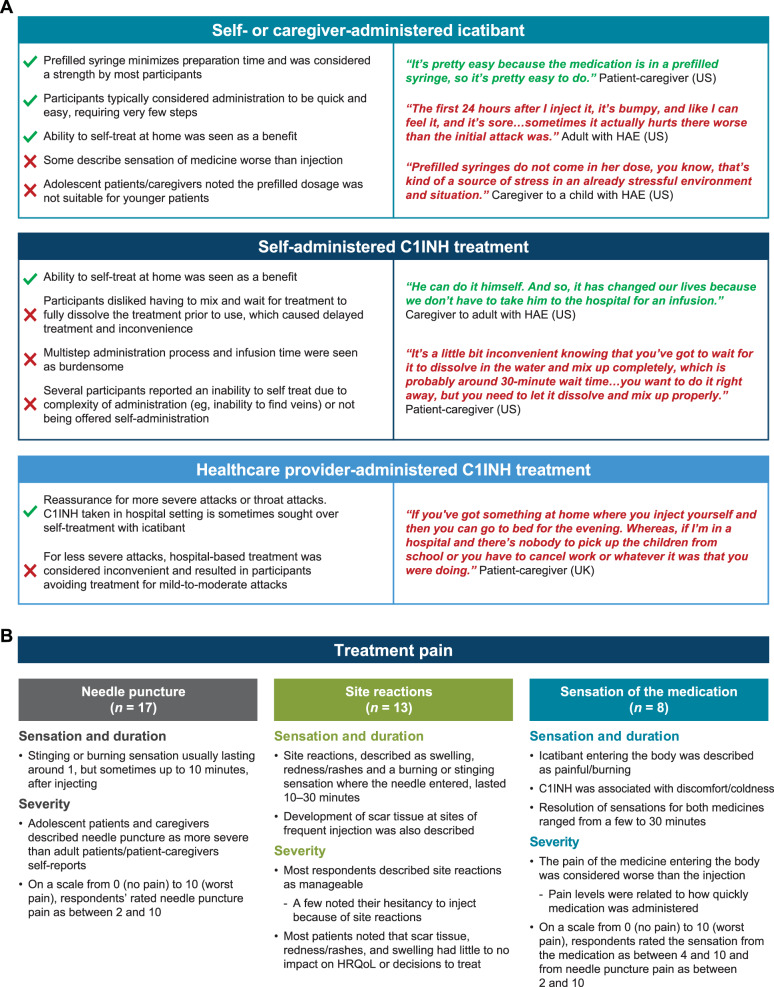
Perceptions of injectable on-demand treatment. **A** Treatment preparation and administration. **B** Pain of injectable treatment. C1INH, C1 inhibitor; HAE, hereditary angioedema; HRQoL, health-related quality of life; UK, United Kingdom; US, United States

**Fig. 4:**
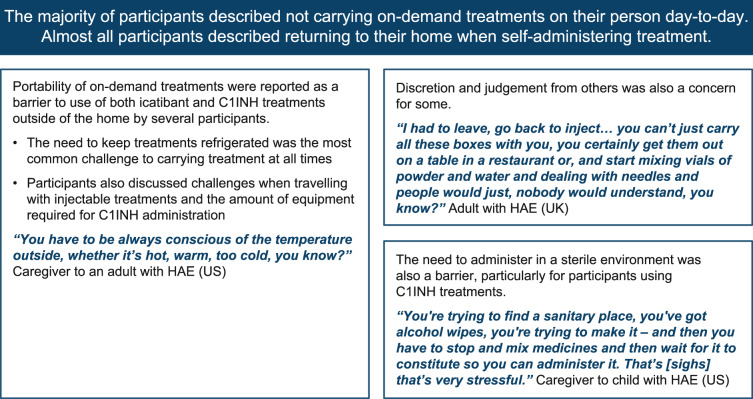
Perceptions of injectable on-demand treatment: barriers to treating outside the home. C1INH, C1 inhibitor; HAE, hereditary angioedema; UK, United Kingdom; US, United States

**Fig. 5:**
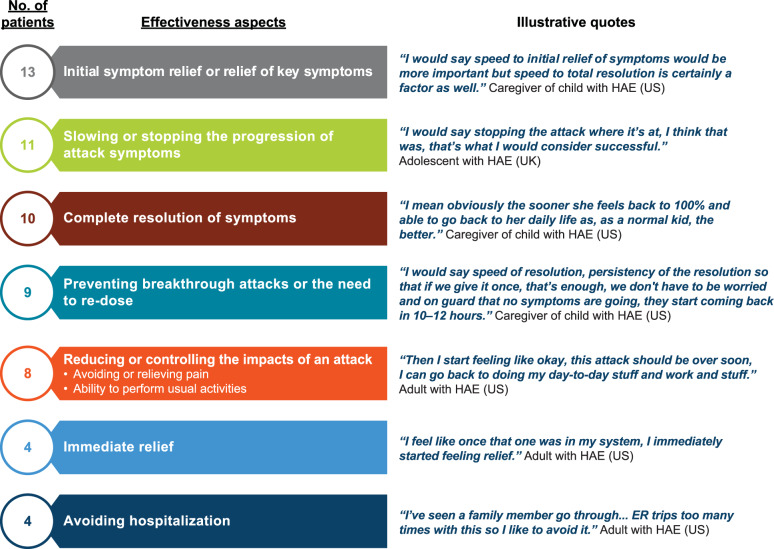
Perceptions of injectable on-demand treatment: defining treatment effectiveness. ER, emergency room; HAE, hereditary angioedema; UK, United Kingdom; US, United States

Participants expressed mixed satisfaction with existing on-demand treatments. All participants described at least one area of dissatisfaction related to treatment preparation, administration, pain, logistical barriers to treating outside the home, and treatment effectiveness (Figs. 3, 4, and 5). Icatibant users reported being generally satisfied with prefilled syringes, which were considered easy and quick to administer (Fig. 3A). Whereas C1INH users were generally less satisfied with treatment preparation—with C1INH therapies perceived as more challenging, with a time-consuming IV administration process—some believed they were unable to self-administer because of the complexity of the process (e.g., applying a tourniquet, finding a vein). Self-administration was generally preferred, but was not possible for many participants, particularly adolescents and C1INH users. Participants who received treatment at medical facilities generally viewed this as burdensome, although some reported it was reassuring compared with self-administration, particularly for more severe attacks.

All participants described the pain and discomfort of injectable treatment (Fig. 3B). Patients were able to distinguish between pain from a needle puncture (*n* = 17), pain related to injection site reactions (*n* = 13), and pain from the sensation of the medicine (*n* = 8), which some rated as more severe than needle punctures (range of pain, 4–10 [where ‘10’ is the worst pain] versus 2–10, respectively). Most participants (9 patients and 5 caregivers) discussed that the lack of portability of these treatments (e.g., need for refrigeration, equipment needed) was a barrier to the use of both icatibant and C1INH outside of their home (Fig. 4). Many patients defined effective treatment as the speed at which initial or key attack symptoms were relieved (*n* = 13), with a small number of patients considering immediate symptom relief (*n* = 4) or avoiding hospitalization (*n* = 4) as important attributes of an effective treatment (Fig. 5). Generally, C1INH therapies were seen as more effective than icatibant and were preferred in acute situations, despite the more complex and inconvenient administration procedures.

### Impacts of injectable on-demand treatments

Participants reported both positive and negative psychological impacts of injectable on-demand treatment, as well as practical and activity limitations, that affected both patients (Table 3) and caregivers (Table 4). Patients expressed gratitude at having access to safe, effective treatments; however, they also described their anxiety and fear associated with these treatments. Patients (*n* = 6) and caregivers (*n* = 6) described having anxiety and worry about administering treatments quickly and correctly to control attack symptoms; these feelings were reported more often in patients self-administering IV treatments, as the preparation and administration process took longer and was more complex. A few patients described receiving treatment in a hospital setting as anxiety inducing because of their discomfort with medical environments. Fear among patients was primarily discussed in relation to needles or the pain they induced, creating a hesitancy to inject on-demand treatments. These fears of hospitals and needles were reported more by the adolescent patients than the adult patients. Rather than fear, caregivers described feeling anxious (*n* = 6) and stressed (*n* = 4) about the responsibility of administering treatments and their care-recipient’s distress during administration. They also reported that the unpredictability of attacks, which could disrupt work and activities at any moment and made them feel constantly ‘on-call,’ exacerbated existing stress and anxiety.

**Table 3:** Impacts of injectable on-demand treatment on patient HRQoL (*n* = 17)

Impact	*n*^a^	Illustrative quotes from patient participants
*Psychological impacts*		
Positive impacts and relief	7	*“It [on-demand treatment] actually lessened my attacks because of all the mental anguish of worrying about it, knowing that I, you know, if anything happened, I had this amazing shot that would stop it. So, I’ve always been like a huge lover of these acute medications.”*** Patient-caregiver (US)**
Anxiety/worry	6	*“I’m a little bit shaky and a little bit nervous when I’m doing it.”*** Adult with HAE (UK)**
Fear	4	*“Since it [needle] was this big, I feel like I would have been panicking a lot because I don’t like needles.”*** Adolescent with HAE (US)**
*Practical and activity limitations*		
Daily or leisure activities	10	*“If I’m 8 h in a hospital and there’s nobody to pick up the children from school or you know, just you, you have to cancel work or whatever it was that you were doing, it’s considerably inconvenient.”*** Patient-caregiver (UK)**
Work or education	7	*“The more portable it is, it might be an option to take it to work with me or something like that or if it’s easy to do, obviously if something happens at work or right before work, I can do it in a timely manner and not have to miss out on missing work.”*** Adult with HAE (US)**
Relationships and family	5	*“It’s a lot not just on me but my family too because they want to make sure that I’m not alone when I do it in case something happens. Knowing that I have to take it, you know, often or inject often, it’s a lot, it’s definitely overwhelming at times.”*** Patient-caregiver (US)**

HAE, hereditary angioedema; HRQoL, health-related quality of life; UK, United Kingdom; US, United States

^a^Counts include prompted and unprompted mentions and only include patient self-reported impacts and not proxy-reported impacts by caregivers

**Table 4:** Impacts of injectable on-demand treatment on caregiver HRQoL (*n* = 8)

Reported impact	*n*^a^	Illustrative quotes from caregiver participants
*Psychological impacts*		
Anxiety/worry	6	*“I’m a nervous wreck, especially praying that it works and it works fast.”*** Caregiver to adult with HAE (US)** “*Whenever they’re [person they care for] stressed that is a stressor for me, so I have to make sure to stay as calm as possible. So, I’ve learned coping methods to be able to administer calmly without a shaking hand.”*** Caregiver of adult with HAE (US)**
Stress	4	*“I’d never injected anyone before and so that was nerve racking.”* ** Caregiver to adult with HAE (US)**
Unpredictability	3	*“I had to leave work, or I would have to go home and give him an injection, so that definitely was sort of you know, as you never know when it will happen, it could be any day, you know? Any time of the day, it could be at night.”* ** Caregiver to child with HAE (UK)**
*Practical and activity limitations*		
Daily or leisure activities	5	*“You know, it’s just, it’s always on our [minds]. Yes, we always have a go bag in the car for you know, with an ice pack but some days you just think, ‘If only we didn’t have to worry about making time for just in case’ or ‘could we do it another way?’”*** Caregiver to adult with HAE (UK)**
Work or education	4	*“Adjusting with work like, ‘Hey I gotta hop off you know on a meeting ‘cause I gotta tend to my spouse for a minute,’ that kind of thing. So, it does require some reshuffling of planned activities.”* ** Caregiver to adult with HAE (US)**
Relationships and family	4	*“Sometimes my husband would get really angry with me because some injections were less painful, some injections were really, really painful.*”** Caregiver to adult with HAE (US)**
Time spent providing emotional support	3	*“… Help them through the mental part of it too ‘cause it’s always scary for them.”* ** Caregiver to adult with HAE (US)**

HAE, hereditary angioedema; HRQoL, health-related quality of life; UK, United Kingdom; US, United States

^a^Counts include prompted and unprompted mentions and only include caregivers’ own self-reported impacts and not proxy-reported impacts by patients

Participants also described frustration at having to stop daily and social activities to administer treatments. Some patients and caregivers described disruptions to leisure activities (*n* = 15) and having to leave work or school (*n* = 11), and these negative impacts were reported more frequently for adolescents. Impact on relationships was reported by patients (*n* = 5) and caregivers (*n* = 4), including patients feeling like a burden to family members and missing social activities because of the need to administer on-demand treatments. Similarly, caregivers reported that the stressful circumstances of administering on-demand treatments impacted their relationships with care-recipients, particularly their partners. A few caregivers described the time spent consoling and supporting care-recipients after administration as another strain related to treatment. Caregivers of children typically reported greater burdens than caregivers of adults. Caregivers who were also diagnosed with HAE (i.e., patient-caregivers) and those who were not reported similar burdens.

### Perceptions of oral treatment

All participants expressed interest in an oral on-demand treatment when asked directly, with some suggesting this spontaneously during interviews while discussing the difficulties with their current injectable treatments. Participants indicated that if they had oral on-demand treatment available, they would treat attacks earlier (*n* = 8).

More than half of participants reported positive perceptions of a potential oral therapy, including dosing flexibility, no preparation, fast and discreet administration, no storage requirements, and increased portability. Most patients and caregivers stated they would prefer oral treatments because they could avoid the pain and emotional impacts of injecting treatments. Most participants considered swallowing to be easier and quicker than injecting treatment, thus potentially helping to avoid attack progression and reducing attack duration.

Perceived disadvantages related to the use of an oral on-demand treatment were also reported. More than half of participants believed the need to swallow a tablet was a potential disadvantage if they were having a throat or facial attack. Others did not perceive this as an issue because they believed they would be able to swallow before their throat felt closed. Some participants expressed concerns about the speed at which an oral tablet would begin to work, as a pill needs to dissolve to enter the blood stream, unlike injectable medications.

When asked to rate HRQoL at the time of the interview (“current HRQoL”), the mean EQ-5D index score for the patients who were included in this task (*n* = 5) was 0.886 (Supplementary Table 2). When asked to imagine having access to an oral on-demand treatment, patients’ mean index scores did not change between the “current HRQoL” and “with availability of an oral treatment” scenarios, despite qualitatively describing how having access to an oral treatment would improve their HRQoL when probed. Patients, in particular, believed an oral treatment would reduce their anxiety, increase ease of administration, and improve ability to carry out usual activities.

## Discussion

This qualitative study is one of only a few that engaged the caregivers of patients with HAE, providing additional insight into the real-world management of this rare genetic condition. To the authors’ knowledge, this is the first study in which patients and their caregivers have distinguished between the different types of pain (i.e., needle puncture versus injection-site reactions versus sensation of the medicine) associated with injectable on-demand treatment. Moreover, this study presents the first qualitative evidence that the burning or pain associated with on-demand treatment can lead to patients not treating attacks early or at all. The key themes emerging from this study echo those of others (e.g., delays in treatment, treatment-related anxiety) [23, 30, 31, 33, 34]. Further, this study highlights the challenges associated with on-demand parenteral treatment administration, including pain and the need for administration assistance; the negative impacts on relationships; and the disruptions to work, school, and leisure activities experienced by both patients and caregivers.

The majority of participants (80%) reported not treating every attack; all participants described delaying treatment intentionally or unintentionally at least once. Although participants generally considered their on-demand treatment to be effective, they noted the need to tolerate the pain and discomfort of administration, as well as the need to overcome issues with portability. However, some did note that the effectiveness of on-demand therapy was reduced when treatment was delayed, illustrating how patients recognize the trade-offs between the pain (severity) of the attack and the pain of injecting treatment, as well as between the impact of the attack on the ability to complete daily activities and the interruption/inconvenience associated with preparing/administering treatment. Responses demonstrated not only how patients assessed attack severity but also highlighted their tolerance for attack severity in relation to conducting activities of daily living. Many patients with HAE report notable work productivity issues [42]; an understanding of how patients balance attack severity with activities of daily living may help guide the use of on-demand treatment in reducing these impairments.

Perceptions and experiences related to treatment administration varied across subgroups. The most common HRQoL impacts centered around (1) disruptions to daily/leisure activities, work, or school and (2) negative psychological impacts, such as anxiety, fear of injection, and stress. Compared with adult patients, adolescent patients reported treating attacks less frequently, a stronger dislike of needle administration, and a greater impact on daily activities—a finding supported by prior assessments of children and adolescents with HAE [43, 44]. These differences may be due, in part, to restricted use of icatibant to adult patients only (age ≥ 18 years) [12] in the US, requiring some adolescents and children to go to a health care facility to receive on-demand treatment. Caregivers of children with HAE reported a greater burden than caregivers of adults with HAE. Although these findings illustrate the burden to caregivers, to our knowledge, no studies have specifically examined the magnified burden faced by patient-caregivers (i.e., those treating their own HAE while caring for someone else with HAE). Our study can potentially serve as a springboard for identifying and addressing the unique challenges faced by patient-caregivers. These findings emphasize that many patients and caregivers do not follow guideline recommendations for carrying and administering on-demand treatment for every attack because of portability issues and the burden of treatment.

All participants expressed an interest in an oral on-demand treatment, describing how an oral treatment would help to overcome the barriers associated with the timely administration of injectable on-demand treatment (i.e., pain and challenges with portability) and reduce treatment burden for patients and caregivers alike.

No change was observed in the mean EQ-5D index scores for “current HRQoL” versus “with availability of an oral treatment,” despite patient interviews eliciting strong beliefs that an oral treatment would improve their HRQoL. This may suggest the EQ-5D is not sensitive to improvements to HRQoL anticipated with an effective oral treatment.

### Limitations

The use of patient advocacy groups for recruitment minimized the likelihood of people without a genuine diagnosis of HAE taking part in this study but may have led to selection bias (i.e., these patients may have been highly motivated). Additionally, most interview participants were female and White. While women tend to have a higher incidence of HAE [2, 45], racial/ethnic breakdowns are less defined, as minority patients tend to be underrepresented, with documented disparities in research and care [46, 47]. In this qualitative analysis, socioeconomic status was similar between US and UK participants as well as participants using on-demand treatments administered by intravenous infusion and subcutaneous injection. Quantitative conclusions cannot be drawn from the qualitative data presented as (1) due to the limited number of patients with HAE, we aimed to address several objectives in a single study, and the interview duration was adapted throughout data collection to meet these objectives; (2) counts may not reflect the importance of themes because the prioritization of questions changed throughout data collection; and (3) some patients were related to caregivers and this was not accounted for in the analysis, meaning the potential of double counting cannot be ruled out.

## Conclusions

Using in-depth interviews to explore the impact of different types of on-demand treatments on patients with HAE and their caregivers, this study confirmed previous reports of the salient barriers related to injectable therapies, e.g., accessibility, portability, and pain. We also uncovered differences in the types of pain associated with injectable on-demand treatments (i.e., from needle puncture, injection site reactions, and sensation of the medicine) and learned that the burning and pain of on-demand treatment may deter patients from treating their attacks in a timely manner or at all. All patients and caregivers described injectable on-demand therapies, including their side effects as having a negative impact on HRQoL. An oral on-demand option may reduce the barriers to early treatment, improve patient independence, and potentially reduce the burden associated with parenteral on-demand treatment for not only patients living with HAE but also their caregivers.

## Supplementary Information

Below is the link to the electronic supplementary material.

Supplementary Material 1

## Data Availability

KalVista Pharmaceuticals accepts requests from qualified researchers who wish to access clinical trial data and associated information, such as clinical study reports with appropriately redacted appendices to protect participant privacy. Please direct your inquiry to DSP@kalvista.com for more details.

## Abbreviations

3L: 3 level
5D: 5 domain
5L: 5 level
C1INH: C1 inhibitor
CE: Concept elicitation
EQ: EuroQol
FDA: United States Food and Drug Administration
HAE: Hereditary angioedema
HAEA: Hereditary Angioedema Association
HAEi: Hereditary Angioedema International
HRQoL: Health-related quality of life
IV: Intravenous
LTP: Long-term prophylaxis
SC: Subcutaneous
VAS: Visual analog scale
UK: United Kingdom
US: United States
