# Supplementary material for: Impact of injectable HAE on-demand treatments on health-related quality of life: a patient and caregiver interview study
Source: Allergy Asthma Clin Immunol. 2025 Nov 29;21:52. doi: 10.1186/s13223-025-00997-w (PMC12715920; doi:10.1186/s13223-025-00997-w)
Supplement: Supplementary file 1 — Supplementary Material 1 [file 13223_2025_997_MOESM1_ESM.docx]

**Additional file 1**

**TABLE S1.** Illustrative quotes from participants on key discussion themes

| **Key themes** | **Illustrative quotes** |
| --- | --- |
| ***Reasons for delaying or forgoing treatment*** |  |
| Access factors | “Insurance problems, you know, I would get attacks like a couple of times a week and at one time, I was only allowed three shots per month, so the only problems were really like insurance wise and having access to them.” **Adult with HAE (US)**  “I haven’t actually used a rescue medication in, I think, a year because I haven’t had time to get the medication filled.” **Adult with HAE (US)**  “I don't have my own supply at home, they allegedly keep it in hospital for me, so when I truck up at the hospital, to A&E, then I have a bit of a battle with the medical staff as to getting them to agree that that’s the treatment that I need, if you like. So sometimes you can be waiting in A&E for, well a couple of hours.” **Adult with HAE (US)** |
| Logistical factors | “A lot of it is also 'cause my mom works and so I don't always have her immediately there to come give me the shot, I have to usually wait until it’s a better time or if her boss is willing to let her off early or just until she is out of work and can get home." **Adolescent with HAE (US)**  “One of the hardest parts is when we don’t have it with us and we have to like rush home to make sure we have it to give it to him." **Caregiver to adult with HAE (US)** |
| Attack features | “Sometimes, um, like even right now my left foot, I feel a little bit of uncomfortableness, swelling but I can tell like it’s not a big deal, it’s not gonna get bad so I’m not doing anything about it." **Patient-caregiver (US)**  “Just depends on the location. I won’t, I won’t take it if it’s in my arms, any-anywhere that I can like, it doesn’t affect my daily life I won’t take it. Therefore, if it affects my face, my intestines, and those are the two. areas I’m like I will take it." **Patient-caregiver (US)**  “I usually wait until I can tell that it’s like an attack and not just me feeling funny, or something weird happening.” **Adolescent with HAE (US)** |
| Treatment features | *“[I] dislike of injecting myself, that’s the main thing that puts me off from taking it. That’s the only reason really why I would delay it at all."* **Adult with HAE (UK)**  “I did not like it, it was very, very painful and it hurt a lot. So, after that, there was moments like with my foot or something, where I would be like, ‘I’ll just hop around a lot’ instead of taking the shot because of just how bad it was.” **Adolescent with HAE (US)**  “It’s a little bit inconvenient knowing that you’ve got to wait for it to dissolve in the water and mix up completely, which is probably around 30-minute wait time. You want to do it right away, but you need to let it dissolve and mix up properly.” **Patient-caregiver (US)**  “Because of the pain of the shot, really. If it’s getting in the way of me writing or like in my schoolwork or if it’s in my lip, it’s getting in the way of talking or eating food, that’s usually when I will get a shot.” **Adolescent with HAE (US)** |
| Site reactions | “The first 24 hours after I inject it, it’s bumpy and like I can feel it and it’s sore. Sometimes it actually hurts there worse than the initial attack was.” **Adult with HAE (US)**  “I get some rash or some sort of swelling, you know, and for a few hours, if I do any activity or walk, I can sort of feel that I have taken an injection. So that’s the thing that I dislike.” **Adult with HAE (UK)** |
| Pain of medication | “I’m going to assume that medication going into my body, it stings, it burns. The actual needle that doesn’t really bother me but feeling the stinging sensation when you’re pushing the medication, yes. Then the area becomes really red and puffy sometimes.” Patient-caregiver describing IV C1INH (US)  “I can’t describe it. All I can say is it’s excruciating pain.” **Patient-caregiver describing SC icatibant (US)** |
| ***Impact of treatment delays*** |  |
| Treatments less effective | “If I delay it, it definitely lengthens the attack and if you delay it a lot, it might not even work, the treatment.” **Patient-caregiver (US)**  “Sooner you take it, the more effective it is, and less likely to relapse and have attacks again. And also less likely that actually at the offset of the pain and sometimes I’ve delayed it too long and I’ve been injecting and the pain’s come and it’s really horrible. So try and inject yourself and go through the pain is pretty bad but you're kind of thinking ‘It’s gonna get worse if I don't do this,’ so you’ve just got to get through it.” **Adult with HAE (UK)** |

*C1INH*, C1 inhibitor; *HAE*, hereditary angioedema; *IV*, intravenous; *SC*, subcutaneous. *UK*, United Kingdom; *US*, United States.

**TABLE S2.** HRQoL: Scenario-based EQ-5D index scores^a^

| **Participants** | **Feelings at the time of the interview, mean (SD)** | **Expectations if there were access to an oral on-demand treatment,  mean (SD)** | **Feelings during their or their care-recipient’s last HAE attack,  mean (SD)** |
| --- | --- | --- | --- |
| Patients (n=5) | 0.886 (0.187)^b^ | 0.886 (0.187)^b^ | 0.447 (0.286)^c^ |
| Caregivers (n=5)^d^ | 0.769 (0.262) | 0.810 (0.329) | 0.507 (0.496) |

^a^All participants except one caregiver were from the US; therefore EQ-5D responses were analyzed using US preference weights to maximize the sample size [Hernández-Alava 2022] and assessed based on US population norms (0.851) [Jiang 2020]. ^b^Mean and standard deviations identical due to exact same responses to EQ-5D instrument for the two scenarios. ^c^One patient was not asked about their last attack as they were currently experiencing an attack. ^d^Caregivers were asked to respond based on their own HRQoL in each scenario, not the person they cared for. *HAE*, hereditary angioedema; *HRQoL*, health-related quality of life; *SD*, standard deviation; *US,* United States.

**Supplementary references**

Hernández Alava M, Pudney S, Wailoo A. Estimating the relationship between EQ-5D-5L and EQ-5D-3L: results from a UK population study. Pharmacoeconomics 2023;41(2):199-207. doi:10.1007/s40273-022-01218-7.

Jiang R, Janssen MFB, Pickard AS. US population norms for the EQ-5D-5L and comparison of norms from face-to-face and online samples. Qual Life Res 2021;30(3):803-16. doi:10.1007/s11136-020-02650-y.
